# Clinical efficacy of Yukgunja-tang combined with a proton pump inhibitor for refractory gastroesophageal reflux disease: study protocol for randomized, double-blind, double-dummy clinical trial

**DOI:** 10.1186/s12906-023-04283-3

**Published:** 2023-12-07

**Authors:** Na-Yeon Ha, Jung-Wook Kim, Jinsung Kim

**Affiliations:** 1 Department of Digestive Diseases, College of Korean Medicine, Kyung Hee University, Seoul, 02447 Republic of Korea; 2 Division of Digestive Diseases, Department of Korean Internal Medicine, Kyung Hee University Korean Medicine Hospital, Seoul, 02447 Republic of Korea; 3 Division of Gastroenterology, Department of Internal Medicine, College of Medicine, Kyung Hee University, Seoul, 02447 Republic of Korea; 4 Division of Gastroenterology and Hepatology, University of Michigan, Ann Arbor, MI 48109 USA

**Keywords:** Gastroesophageal reflux disease, Herbal medicine, Botanical drug, Yukgunja-tang, Liujunzi-tang, Rikkunshito, Proton pump inhibitor, Rabeprazole

## Abstract

**Background:**

Yukgunja-tang (YGJ) is an herbal prescription used to treat the symptoms of gastroesophageal reflux disease (GERD). Although many preclinical and clinical studies on YGJ have been conducted on GERD, there is a lack of evidence from blinded studies to exclude placebo effects. Therefore, this protocol proposes a clinical trial that is single-centered, randomized, double-blinded, double-dummy to objectively evaluate the efficacy and safety of co-administered YGJ and rabeprazole (RPZ) in patients with GERD previously treated with proton pump inhibitors (PPIs) and still experiencing symptoms.

**Methods:**

A total of 86 participants with refractory GERD (rGERD) will be randomized in a 1:1 ratio to the treatment [YGJ and RPZ (10 mg/d)] and control groups [double-dose RPZ (20 mg/d)] for 4 weeks of treatment (weeks 0–4) followed by 4 weeks of follow-up (weeks 4–8). The Frequency Scale for the Symptoms of GERD will be analyzed for the primary endpoint. Reflux Disease Questionnaire, Reflux Symptom Score, GERD-Health Related Quality of Life, Overall Treatment Evaluation, Spleen Qi Deficiency Questionnaire, Damum Questionnaire, and dyspepsia Visual Analogue Scale will be used to evaluate treatment effects on GERD related symptoms and quality of life and to compare treatment effects by subgroups. Safety tests will be analyzed by investigating adverse events.

**Discussion:**

This clinical trial will be the first rigorous double-blind, double-dummy, placebo-controlled study to precisely evaluate the efficacy and safety of the combination of YGJ and PPIs in the treatment of rGERD. The results of this study will provide a reliable clinical basis for selecting botanical drug treatments for patients with rGERD.

**Trial registration:**

Clinical Research Information Service (registration number: KCT0008600, July 13, 2023, https://cris.nih.go.kr).

## Background

Gastroesophageal reflux disease (GERD) is a condition in which stomach contents flow back into the esophagus or oral cavity, causing discomfort or complications [1]. Typical symptoms of GERD include heartburn or acid regurgitation. Extraesophageal symptoms may include chest pain, coughing, and hoarseness. These symptoms can worsen with weight gain [2–5]. The prevalence of GERD varies worldwide, ranging from 2.5 to 33.1%. Since 1995, there has been a rising trend in its incidence, particularly in North America and East Asia [6]. Consequently, there is a growing interest in its ongoing treatment and management due to the societal cost associated with reduced quality of life [7, 8]. Standard treatments for GERD include weight loss, proton pump inhibitors (PPIs) like omeprazole, rabeprazole (RPZ), and esomeprazole, histamine-2 receptor antagonists (H2RAs), prokinetics, and occasionally anti-reflux surgery [4, 5].

GERD is categorized into erosive esophagitis (EE) and non-erosive reflux disease (NERD) based on the presence or absence of esophageal mucosal damage on endoscopy. A study showed that the response rates to 4 weeks of PPI therapy were 37% for NERD and 56% for EE, indicating lower effectiveness in patients with NERD [9]. GERD often follows a chronic course with symptoms recurring upon treatment discontinuation. Thus, effective treatments and lifestyle modifications are crucial [10]. The causes and mechanisms of GERD symptoms vary, leading to cases where PPIs do not yield improvement, known as refractory GERD (rGERD) [11]. Patients unresponsive to once-daily PPI therapy might benefit from twice-daily dosing and long-term maintenance therapy [12]. Although long-term PPI use is essential for GERD, concerns about associated adverse effects, such as *Clostridium difficile* infection, bone fractures, and acute kidney injury, have been raised [13, 14]. rGERD involves diverse pathophysiologies, including acid nature, reflux, delayed gastric emptying, motility inefficiencies, and cytochrome P450 (CYP) isoenzyme, CYP2C19, genetic polymorphism. To alleviate symptoms, a combination of PPIs and prokinetic agents is often used [15–19]. Complementary and alternative therapies, including botanical drugs, are actively researched as promising GERD management options [20]. If the therapeutic effects of these botanical drugs on GERD are verified, they could potentially reduce overall PPI dosage when used alongside or as an alternative to PPIs.

Yukgunja-tang (YGJ in Korean, Liujunzi-tang in Chinese, Rikkunshito in Japanese) is a prescription comprising eight botanical drugs: Ginseng Radix [*Araliaceae*], Atractylodis Rhizoma Alba [*Compositae*], Poria Sclerotium [*Polyporaceae*], Pinelliae Tuber [*Araceae*], Citri Unshius Pericarpium [*Rutaceae*], Zizyphi Fructus [*Rhamnaceae*], Glycyrrhizae Radix et Rhizoma [*Leguminosae*], and Zingiberis Rhizoma Recens [*Zingiberaceae*]. Its prokinetic and gastric mucosa protective effects have been utilized in East Asia to treat conditions like gastritis, functional dyspepsia (FD), and GERD, often characterized by symptoms such as loss of appetite and dyspepsia [21–27]. In Japan, a combination of conventional medicine and botanical drugs for rGERD has been widely prescribed due to its reported efficacy and safety. It is also included as a treatment option in GERD clinical practice guidelines. YGJ has been shown to modulate impaired gastric accommodation and delayed gastric emptying, making it potentially more effective in alleviating GERD symptoms, especially in patients with dyspeptic symptoms. Studies have demonstrated that YGJ, used alone or in combination with PPIs, effectively alleviates reflux symptoms in patients with rGERD [24, 28–32]. Despite these studies, as far as we know, no randomized, double-blind, double-dummy, placebo-controlled studies using a double placebo for either medicine have been conducted to provide clinical evidence for co-administration of YGJ and PPI as a treatment option for patients with GERD. Therefore, if the efficacy and safety of the combination treatment are proven, it will be possible to maximize the effectiveness of combination therapy. This can be achieved by reducing the recurrence rate of GERD and minimizing the side effects caused by long-term PPI administration.

The goal of this study is to propose a clinical trial protocol to objectively validate the efficacy and safety of a combination of an extract mixture of botanical drugs (YGJ) and a PPI (RPZ) in patients with GERD who have been treated with PPIs in the past and currently have residual symptoms.

## Methods

### Study design

This study is a single-center, randomized, double-blind, double-dummy, parallel-design clinical trial to evaluate the safety and efficacy of co-administered YGJ and PPI for rGERD. Patient recruitment has already commenced in August 2023 and will continue until February 2025 at the Kyung Hee University Korean Medicine Hospital (Seoul, Republic of Korea).

A total of 86 participants who fulfill the study criteria and consent to participate will be randomized in a 1:1 ratio for the test (43 participants; YGJ and RPZ 10 mg/d) and control (43 participants; RPZ 20 mg/d) groups. The study will consist of a screening visit and 4 weeks of treatment (weeks 0–4), followed by 4 weeks of follow-up (weeks 4–8).

In this study, the principal investigator (PI) and researchers will follow lawful procedures based on ethical principles, including the principles of the Declaration of Helsinki, to provide participants with detailed study information and obtain informed consent for participation. Participants are free to withdraw from the study at any time for any reason.

**Fig. 1:**
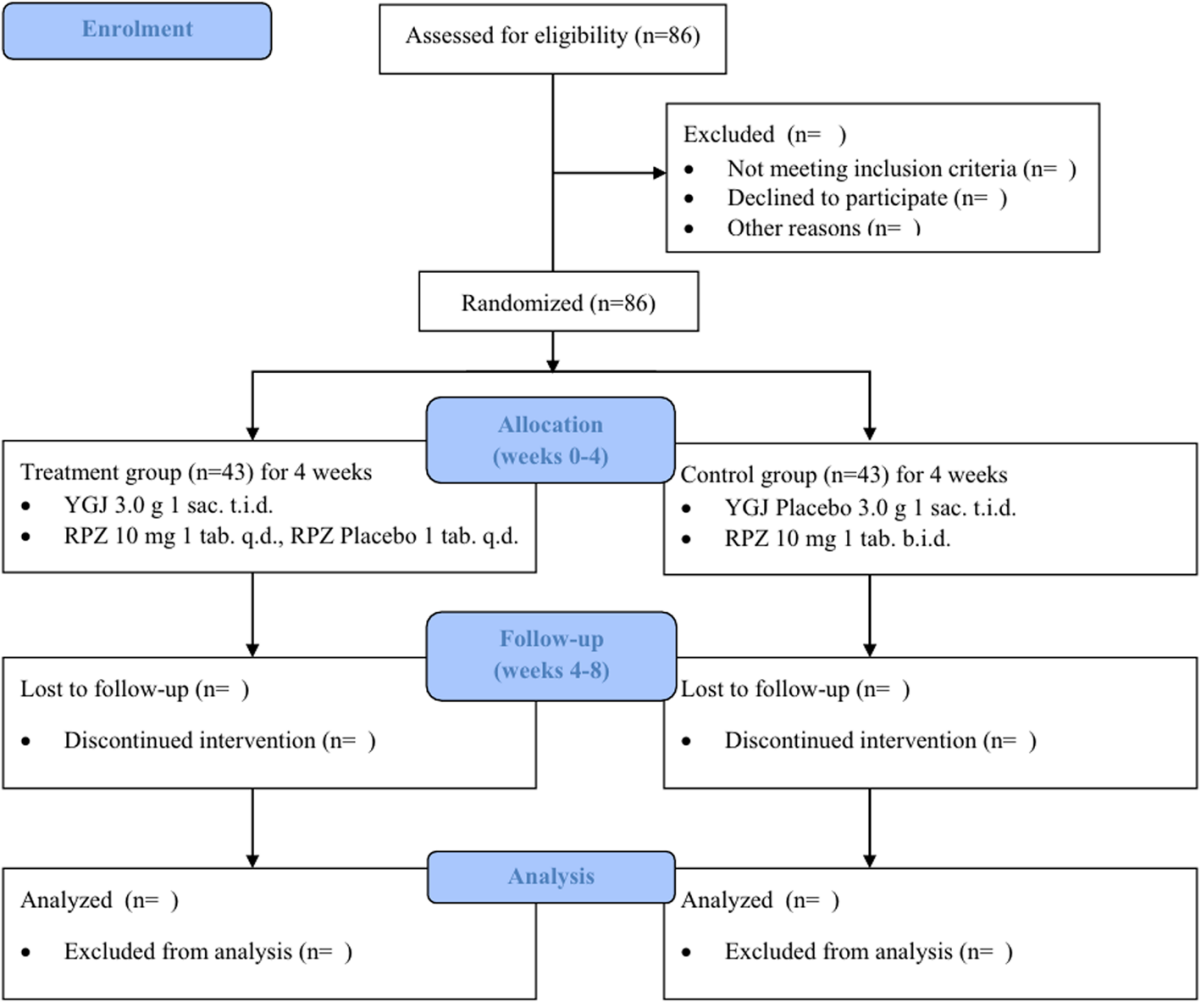
Flow diagram of the trial. B.i.d: Bis in die; RPZ: Rabeprazole; Sac: Sachet; Tab: Tablet; T.i.d: Ter in die; Q.d: Quaque die; YGJ: Yukgunja-tang

This study will be conducted according to an approved protocol by the Institutional Review Board (IRB) of Kyung Hee University Korean Medicine Hospital and will adhere to the Good Clinical Practice standards established by the Korean Food and Drug Administration. The study will also follow the Standard Protocol Items: Recommendations for Interventional Trials (SPIRIT) and the Consolidated Standards of Reporting Trials (CONSORT) statement [33, 34]. The flowchart and outcome evaluation points for this trial are shown in Fig. 1 and Table 1.

**Table 1:**
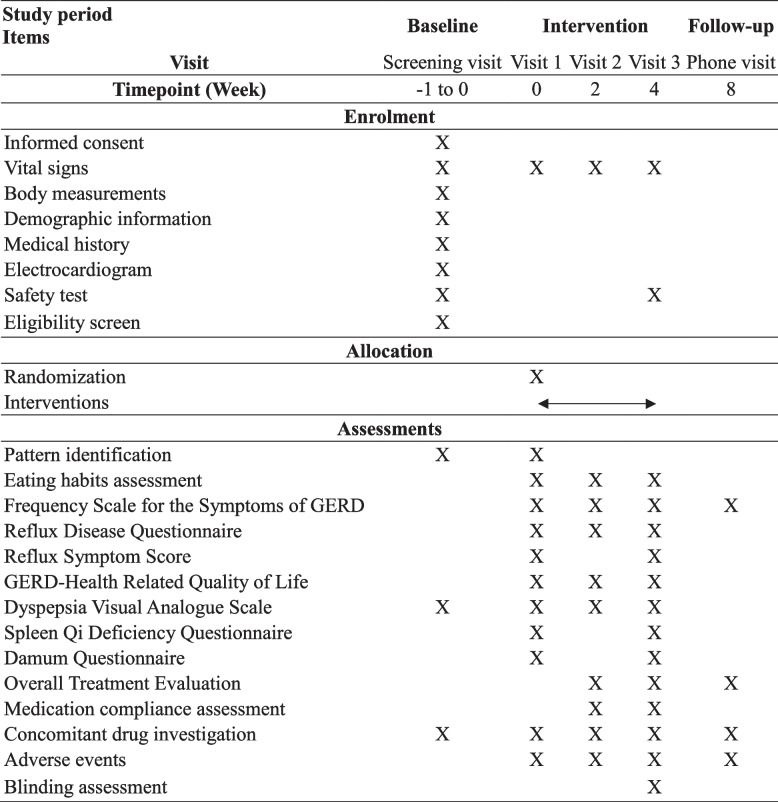
Study procedure of enrolment, allocation, and outcome assessments

*GERD* Gastroesophageal reflux disease

### Participants

#### Recruitment

Participants will be recruited through notices posted on clinical bulletin boards, online platforms, and public transportation bulletin boards.

#### Inclusion criteria

1. Participants aged between 19 and 75 years.
2. Participants diagnosed with GERD, including EE or NERD.
3. Participants who have received PPI therapy for at least 4 weeks but still experience typical GERD symptoms (heartburn and acid regurgitation).
4. Participants with dyspeptic symptoms, measured on a 0–100 mm visual analogue scale (VAS), of 40 points or higher.
5. Participants who voluntarily agree to participate in this clinical trial and have signed the consent form.

#### Exclusion criteria

1. History of hypersensitivity reactions (e.g., rash, urticaria, and itching) to components of the investigational product (IP) or benzimidazole.
2. Participants with the following conditions detected by upper endoscopy: gastric ulcers (excluding scars), ulcer stenosis, esophageal varices, eosinophilic esophagitis, gastrointestinal bleeding, Zollinger-Ellison syndrome, Barrett's esophagus, esophageal stricture, or malignant tumors of the gastrointestinal tract.
3. Participants diagnosed with FD, esophageal motility disorders, irritable bowel syndrome, or inflammatory bowel disease for the primary symptom.
4. Participants presenting warning symptoms suggestive of malignancy (e.g., unintentional weight loss, recurrent vomiting, dysphagia, hematemesis, and melena).
5. Participants who have undergone surgeries affecting gastric acid secretion, such as esophageal surgery, anti-reflux surgery, and gastrectomy.
6. Participants with a history of malignancy within 5 years before the screening date, except for cases other than gastrointestinal malignancies that had been cured without recurrence for at least 5 years from the date of screening.
7. Participants diagnosed with severe liver disease, chronic kidney disease, or any severe renal dysfunction.
8. Participants who have taken specific medications within 2 weeks of receiving IP or need continuous administration of these medications for the treatment of other diseases (including antipsychotics, non-steroidal anti-inflammatory drugs, corticosteroids, etc.).
9. Pregnant or lactating participants.
10. Female participants of reproductive age expected to become pregnant during the clinical trial period.
11. Participants with a history of alcohol addiction or drug abuse.
12. Participants with specific genetic disorders (e.g., galactose intolerance, Lapp lactase deficiency, and glucose-galactose malabsorption).
13. Participants currently receiving Atazanavir or Rilpivirine.
14. Chronic conditions poorly controlled despite appropriate treatment (e.g., hypertension, diabetes, and edema), posing a threat to life, requiring hospitalization, leading to significant physical disabilities, or interfering with daily activities.
15. Participants who have participated in another clinical trial within 30 days prior to the date of written consent.
16. Participants who have taken herbal/traditional medicine products or received traditional medicine treatments (e.g., acupuncture and moxibustion) affecting gastric acid secretion or the gastrointestinal system within 2 weeks prior to IP administration.
17. Participants unable to participate or receive treatment in the clinical trial due to severe mental disorders.
18. Participants with clinical abnormalities identified through screening tests (vital signs, blood and urine tests, and electrocardiogram).
19. Participants deemed unsuitable for participation in the study by the investigator.

### Randomization and allocation concealment

Randomization will be assigned to each arm by a statistician blinded to the conduct and evaluation of the study. This will be based on a pre-generated 86-person (1:1) random number table using the statistical programs such as SAS® Version 9.4 (SAS Institute Inc., Cary, NC, USA) or IBM SPSS Version 25.0 (IBM Corp., Armonk, NY, USA). Each patient's allocation, according to the randomization table, will be placed in an opaque envelope, sealed in a double envelope, and stored until the end of the study. The random number will be communicated to the IP manufacturer's representative, who will mark the IP on the label without differentiation by group. Participants providing written consent and meeting all inclusion criteria will be enrolled and assigned a random number by the clinical research coordinator in the order of their visit.

### Blinding

The trial will be double-blinded by producing placebos that are externally indistinguishable for both YGJ and RPZ. The manufacturer will package the IP identically to conceal the intervention allocation and affix the randomization number to the IP container and its placebo according to the pre-designed randomization table to ensure double-blinding. Participants, investigators, pharmacists, and outcome assessors will not know which container contains the study drug or placebo, nor will they know the patient's arm assignment.

### Interventions

Participants will be randomized to receive the investigational drugs as prescribed for 4 weeks. The botanical drug preparation of YGJ and its placebo were manufactured by the National Institute for Korean Medicine Development (Gyeongsan, Republic of Korea), and RPZ and its placebo by KMEDIhub (Daegu, Republic of Korea). Each was manufactured in accordance with Korean Good Manufacturing Practice guidelines.

#### Botanical drug and placebo production

##### YGJ

The raw materials of YGJ extract granules per sachet included 1.33 g each of Ginseng Radix (Korean Pharmacopoeia, KP), Atractylodis Rhizoma Alba (KP), Poria Sclerotium (KP), Pinelliae Tuber (KP), and Citri Unshius Pericarpium (KP), 0.67 g each of Zizyphi Fructus (KP) and Zingiberis Rhizoma (Korean Herbal Pharmacopoeia, KHP), and 0.50 g of Glycyrrhizae Radix et Rhizoma (KP). The dry YGJ extract (1.06 g) was mixed with 0.50 g of lactose hydrate (KP) and 1.44 g of corn starch (KP), which was dried to a granular form to obtain 3.0 g of brown YGJ extract.

##### YGJ placebo

The YGJ placebo was formulated considering the color, odor, and taste to minimize external differences from the actual drug for participant and investigator blinding. The optimal prescription was determined by conducting disintegration tests to establish stability during storage and checking the properties of the granules. The placebo (3.0 g/sachet) does not contain the main ingredient effective for the treatment of GERD and consists of lactose hydrate (KP), corn starch (KP), cacao color, caramel color, gardenia yellow color, and ginseng flavor powder.

Both the YGJ and placebo were hermetically packaged in opaque containers and labeled identically. Patients assigned to either treatment or comparison arm will consume one sachet (3.0 g) of YGJ extract granules or a placebo three times a day before or between meals for 4 weeks.

#### PPI and placebo production

##### RPZ

RPZ is a pale yellow-pink, round, enteric-coated tablet containing 10.0 mg of rabeprazole sodium, administered orally as a single tablet.

##### RPZ placebo

The RPZ placebo contains no active ingredients and was produced with RPZ tablet-like appearance, containing only excipients, glossing and coating agents, and purified water.

The test group will take one RPZ tablet (30 min before breakfast) and one placebo tablet (30 min before dinner) per day, whereas the control group will take one RPZ tablet orally twice daily (30 min before breakfast and dinner).

The interval between the dosing of YGJ, RPZ and their corresponding placebos was set to at least 30 min to minimize drug interactions.

#### Medication compliance assessment

Participants in each group will receive IP at visits 1 (week 0) and 2 (week 2). The investigator will check the number of remaining medications to ensure that the participant had taken the medication as directed (visits 2 and 3). The medication compliance rate (%) will be calculated every 2 weeks by dividing the number of medications actually taken by those that should have been taken, with a threshold of 80% for efficacy analysis.

### Concomitant drug investigation

During the study period, the participants are prohibited from taking medications that may affect gastric acid secretion and the digestive system, as determined by the investigator, including those mentioned in point 8 of the exclusion criteria. Patients will be able to participate in this study after discontinuing these treatments prior to study entry, following a 2-week washout period. Additionally, patients are prohibited from receiving other treatments stated in point 16 of the exclusion criteria to relieve the symptoms of GERD during the study. At each visit during the observation period, participants will be instructed to report concomitant medications and contraindications. All concomitant medications and treatments will be recorded in the Case Report Form (CRF).

### Eating habits assessment

During the study period, all participants will be monitored for changes in dietary habits using a food recorder to determine the appropriateness of eating habits concerning dietary factors and foods at risk for GERD (e.g., coffee, alcohol, and fried foods).

### Outcome measures

#### Demographic and baseline information

Demographic information, medical history including medications and surgery, and alcohol consumption will be examined in detail. The GERD-related information will be investigated to determine the timing of symptom onset and diagnosis, upper endoscopy findings, symptom severity, and treatment history. Participants will also be categorized into five subtypes using a 32-item questionnaire of standard tools for the pattern identification of GERD based on their GERD-related symptoms and health conditions: liver-stomach disharmony, middle qi deficiency counterflow, qi depression phlegm obstructing, stomach yin insufficiency, and spleen-stomach dampness-heat patterns [35].

#### Primary outcome

##### Frequency Scale for the Symptoms of GERD (FSSG)

In addition to the typical symptoms of GERD, the FSSG includes an assessment of dyspeptic symptoms, such as bloating and postprandial fullness. Participants will be required to rate how often they experience each of the 12 symptoms on a scale of 0 (never) to 4 (always), with a total of 48 points, which will be summed by dividing the symptoms into two categories: acid reflux and dysmotility [36]. The FSSG will be measured at all visits except the screening visit, and the change in the FSSG score from baseline to 4 weeks will be calculated. The improvement between the baseline and follow-up period will be compared between the groups to determine the persistence of the treatment effect. In addition, the proportion of participants with no GERD symptoms at one-week recall, referred to as a treatment responder, will be compared between the groups.

#### Secondary outcomes

##### Reflux Disease Questionnaire (RDQ)

Gastrointestinal symptoms associated with GERD will be assessed for both frequency and severity of discomfort. Scores will be summed on a Likert-type scale ranging from 0 to 5 with three subscales: heartburn, regurgitation, and dyspepsia [37]. Patients will provide their completed RDQ at visit 1 (week 0), 2 (week 2), and 3 (week 4).

##### Reflux Symptom Score (RSS)

For extra-esophageal throat symptoms and abdominal, chest, and respiratory symptoms caused by acid reflux, the overall severity of symptoms, frequency of occurrence, and impact on daily life are summed using a Likert-type score ranging from 0 to 5 [38]. The RSS will be obtained at visits 1 (week 0) and 3 (week 4).

##### GERD-Health Related Quality of Life (GERD-HRQL)

The impact of GERD symptoms on quality of life, including sleep and eating habits, will be assessed using a Likert-type scale ranging from 0 to 5 [39]. The GERD-HRQL assessment will be conducted at visits 1 (week 0), 2 (week 2), and 3 (week 4).

##### Overall Treatment Evaluation (OTE)

The OTE indicates the patients’ impressions of their treatment, including changes in their overall life and their GERD symptoms during the treatment period. Patients will rate their OTE on a 15-point scale ranging from ‘exacerbated symptoms (-7)’ to ‘no change (0)’ or ‘no symptoms (+ 7)’ [37]. The OTE will be performed at visits 2 (week 2) and 3 (week 4) and the follow-up phone visit (week 8).

##### Spleen Qi Deficiency Questionnaire (SQDQ)

The SQDQ is a 5-point questionnaire that assesses 11 items, including symptoms such as dyspepsia, lethargy, and loss of appetite, to determine dysfunction in the immune and digestive systems [40]. The SQDQ will be administered before and after taking the IP [visits 1 (week 0) and 3 (week 4)], with a cut-off value of 43.18.

##### Damum Questionnaire (DQ)

The DQ consists of 14 items, including dyspepsia, dizziness, and foreign body sensation in the throat, to diagnose phlegm-fluid retention (Damum in Korean), a sticky and murky pathological product of the body [41]. The DQ will be administered before and after taking the IP [visits 1 (week 0) and 3 (week 4)]. Each item is rated on a 7-point scale according to severity, with higher scores indicating the presence of Damum.

##### Dyspepsia VAS

At all visits except the phone visit (week 8), the participants will rate the intensity (ranging from none to very severe) of their dyspepsia symptoms in the past week using a 100 mm horizontal line.

#### Safety outcomes

To assess safety, all patients will be monitored for clinical changes in vital signs (blood pressure, heart rate, and body temperature) at each visit. After receiving IP at visit 1 (week 0), patients will be monitored for any adverse events that occur during the study and the investigator will record their timing, severity, outcome, and causality. In the event of a serious adverse event, such as death, illness requiring hospitalization, or disability, the investigator must promptly take appropriate action based on the pre-arranged insurance process, after the PI has viewed the sealed randomization. Detailed information will be documented and reported to the IRB and the clinical research associate. Blood tests, urinalysis, and pregnancy tests (Urine HCG) will be performed at the screening visit and visit 3 (week 4): common blood count, differential count, aspartate transaminase, alanine aminotransferase, gamma-glutamyl transpeptidase, alkaline phosphatase, lactate dehydrogenase, total bilirubin, direct bilirubin, albumin, blood urea nitrogen, creatinine, total cholesterol, triglyceride, glucose, creatine kinase, sodium, potassium, chloride, magnesium, urinalysis (occult blood, bilirubin, urobilinogen, keton, protein, nitrite, glucose, pH, specific gravity, and leukocyte), and electrocardiogram.

### Sample size calculation

The results of previous study were used to determine the non-inferiority margin required to calculate the sample size [42]. The upper limit of the 95% confidence interval for the mean difference in the degree of improvement of FSSG between the test and control groups after 4-week treatment was estimated to be -8.29. Assuming a significance level of 2.5%, a statistical power of 90%, and a 1:1 allocation ratio, 86 participants (43 per group) would be required to account for a dropout rate of 30%, considering the larger standard deviation of 9.8.

### Statistical analysis

Statistical analyses will be performed by an independent statistician using the statistical software IBM SPSS Version 25.0 (IBM Corp.). The study will use per-protocol set (PPS) as the primary method of analysis, with full analysis set (FAS) analysis as secondary confirmation, if necessary. The PPS analysis will be applied to patients who are fully compliant with the protocol. In this study, data from patients who comply with IP (≥ 80%), assess the primary efficacy endpoint, and complete the study without major protocol violations will be included in the analysis. The FAS population will consist of data from patients who meet the inclusion criteria, have taken the IP at least once, and have at least one endpoint measurement. The safety analyses set will include all data from patients who received at least one dose of IP after randomization.

For the comparative evaluation of demographic or clinical characteristics between groups, two-sample t-tests will be performed for continuous data, and the chi-squared test or Fisher's exact test will be used for dichotomous data. If significant differences are found between the groups, one-way analysis of covariance will be conducted for continuous data, and logistic regression will be performed for dichotomous data to correct the imbalance.

The basic principle of statistical analysis for the efficacy endpoints is the comparison of data between the two groups after the 4-week treatment intervention. For the efficacy endpoint at week 4, a one-sided 97.5% confidence interval will be calculated to confirm non-inferiority (*p* < 0.025) by determining the difference in mean changes of the questionnaire score between the treatment and control groups. All measurements will be presented as mean ± standard deviation or as percentages (n %). Mean comparisons between groups will be conducted using a two-sample t-test as a parametric method or a Mann–Whitney test as a non-parametric method. Differences within each group between baseline and measurements after a specific period will be analyzed using either the paired t-test or the Wilcoxon signed-rank test. Repeated measures will be analyzed to compare the changes in the mean value of the same variable measured over time between the two groups. For safety assessments, the incidence of adverse and serious adverse events will be calculated and tested by group using the chi-square test or Fisher's exact test. Differences in effectiveness between subtypes, where applicable, will be determined by analysis of variance, considering participant characteristics (such as sex and age), GERD classification (including EE and NERD), previous PPI therapeutic dosage, GERD pattern identification, and other clinical characteristics.

### Data collection and monitoring

To maintain the accuracy and quality of the study procedures, data monitoring will be done by HelpTrial Inc. (Seoul, Republic of Korea), a clinical research organization. Thoroughly trained clinical research associates will conduct thorough and regular monitoring to ensure patient consent, detection of adverse events, and adherence of the study in accordance with standard operating procedures.

To ensure accurate and transparent data collection, management, and quality assurance, this study will utilize the electronic data capture system to implement an electronic CRF. Access to the database will be restricted to trained and authorized investigators. Once all the data has been collected, it will be encrypted and delivered to an independent statistician.

## Discussion

To our knowledge, many clinical studies have shown the substantial efficacy of YGJ and PPIs in relieving GERD symptoms safely. However, no double-blind studies have been conducted on combination therapy with YGJ and PPI using each placebo in patients with rGERD. Therefore, this study aims to verify the non-inferiority of combination treatment compared to conventional rGERD treatment using a double-dummy design.

Sample data from the Health Insurance Review and Assessment Service on patients with GERD in South Korea showed that a substantial proportion of patients with GERD were taking PPIs for at least 12 weeks, with the prevalence and prescribed daily dose of PPIs gradually increasing, combined with high medical costs [43].

Nowadays, there is no consensus regarding the diagnostic criteria for rGERD [44]. Although there may be differences in the definitions regarding the dosage and duration of antacids and how to assess symptomatic improvement, approximately 45% of patients with GERD are partially or completely unresponsive to PPIs, and there is a need to develop more effective treatments [45]. Therefore, the definition of "refractory" in this study includes patients who have been treated with PPIs in the past but have not responded adequately to standard short-term PPI therapy, including those who 1) have recurrent symptoms, 2) have been on maintenance therapy for a period of time, and 3) are taking medication as needed for recurrent symptoms.

Based on the opinions of gastroenterologists, safety reports in previous studies, and clinical utility, RPZ was selected as the PPI for use in this trial. Based on previous research articles published in Japan and drug information registered with the Ministry of Food and Drug Safety, the initial dose for symptomatic relief of GERD (including both EE and NERD) in this study was set at 10 mg/day of RPZ [24, 32]. In addition, we confirmed the clinical utilization of the PPI twice daily oral dosing strategy according to the guidelines for patients with poorly controlled GERD and set it as a control group for the combination of botanical drugs [5].

YGJ, one of the representative botanical drugs, is widely used to treat patients with GERD and several experimental and clinical studies have been conducted to investigate the underlying mechanisms on GERD. As a result, its pharmacological effects on motor function throughout the upper gastrointestinal tract similar to those of prokinetic agents have been reported, including the improvement of delayed esophageal clearance and gastric emptying, modulation of visceral hypersensitivity, and reduction of gastric contents and reflux volume [25, 27, 46–49]. These pharmacological properties may explain the mechanism by which YGJ alleviates symptoms in patients with rGERD, especially accompanied by dyspepsia.

In this study, the 4-week study duration is not a continuation of pre-treatment, but rather a treatment duration that is commonly used in clinical practice to evaluate treatment response in symptomatic groups that do not respond efficiently to pre-treatments. Previous studies have shown that 28.5% of patients with GERD did not respond to 4 weeks of initial PPI therapy [50]. At least 4 weeks of continuous PPI therapy is required for the initial treatment of GERD, and most clinical studies measure effectiveness by symptom relief after 4 weeks of PPI therapy [9, 51].

Assessment and quantification of GERD symptoms are important endpoints for efficacy validation. In this study, the FSSG score was selected as the primary outcome by expert consensus to assess the severity and frequency of heartburn and acid reflux, which are typical symptoms of GERD, and to evaluate responders. Based on a previous study that used the FSSG as a primary outcome, we calculated the optimal sample size for hypothesis testing [42]. RDQ, RSS, and dyspepsia VAS were selected as secondary variables to evaluate the specific discomfort of typical and atypical GERD symptoms simultaneously, and GERD-HRQL, a disease-specific questionnaire, will be used to evaluate the quality of life in patients with GERD. In traditional medicine, the indications for YGJ include the patterns of Spleen Qi Deficiency and Damum, characterized by symptoms of general weakness, dyspepsia, and throat discomfort [40, 41]. Thus, the SQDQ and DQ were evaluated to assess the symptom improvement effects of the combination treatment and to compare the differences in efficacy across subtypes. The OTE was selected as an assessment tool to evaluate the patient's impression of the treatment for overall symptom changes, which is most important in the clinical field.

In conclusion, the primary purpose of this study is to clinically confirm the feasibility of substituting YGJ for dose escalation of PPIs. This substitution will serve as a basis for the retrospective exploration of its mechanisms of action and be considered as a treatment option, other than PPIs, to improve GERD symptoms. One limitation of this study is that it is a single-center study specific to Koreans; therefore, it may be difficult to generalize the results to other populations worldwide. However, as the first double-blind, randomized clinical trial to evaluate the efficacy and safety of a cooperative and integrated approach of both Western and botanical drugs in GERD treatment, the results of this study will clarify and strengthen the objective clinical evidence for the effectiveness of combination therapy for rGERD. By expanding the effective treatment options and management methods for GERD through the use of botanical drugs, this approach can contribute to the development and implementation of clinical guidelines. These guidelines will provide valuable medical information to healthcare professionals and help improve public health.

## Trial status

Participant recruitment is currently underway. Eight participants were recruited on October 3, 2023, and enrolment will be completed by February 28, 2025.

## Data Availability

Data sharing is not applicable to this article as no datasets were generated or analyzed during the current study. The results of this trial will be published in peer-reviewed journals in accordance with CONSORT guidelines and disseminated through international conference posters.

## Abbreviations

CONSORT: Consolidated Standards of Reporting Trials
CRF: Case Report Form
CRIS: Clinical Research Information Service
CYP: Cytochrome P450
DQ: Damum Questionnaire
EE: Erosive Esophagitis
FD: Functional Dyspepsia
FSSG: Frequency Scale for the Symptoms of Gastroesophageal reflux disease
GERD: Gastroesophageal Reflux Disease
GERD-HRQL: GERD-Health Related Quality of Life
H2RA: Histamine-2 Receptor Antagonist
IP: Investigational Product
IRB: Institutional Review Board
KP: Korean Pharmacopoeia
KHP: Korean Herbal Pharmacopoeia
NERD: Non-erosive Reflux Disease
OTE: Overall Treatment Evaluation
PI: Principal Investigator
PPI: Proton Pump Inhibitor
RDQ: Reflux Disease Questionnaire
rGERD: Refractory Gastroesophageal Reflux Disease
RPZ: Rabeprazole
RSS: Reflux Symptom Score
SPIRIT: Standard Protocol Items: Recommendations for Interventional Trials
SQDQ: Spleen Qi Deficiency Questionnaire
VAS: Visual Analogue Scale
YGJ: Yukgunja-tang
